# ‘It is not going to change his life but it has picked him up’: a qualitative study of perspectives on long term oxygen therapy for people with chronic obstructive pulmonary disease

**DOI:** 10.1186/1477-7525-11-124

**Published:** 2013-07-25

**Authors:** Juliet Goldbart, Abebaw Mengistu Yohannes, Ryan Woolrych, Susan Caton

**Affiliations:** 1 Research Institute for Health & Social Change, Health Professions Department, Manchester Metropolitan University, Hathersage Road, Manchester M13 8XR, UK; 2 Gerontology Research Centre, Simon Fraser University, Vancouver, BC V6B 5K3, Canada; 3 Research Institute for Health & Social Change, Manchester Metropolitan University, Hathersage Road, Manchester M13 8XR, UK

**Keywords:** Chronic obstructive pulmonary disease, COPD, Long term oxygen therapy, Patient perspectives, Carers, Healthcare professionals, Compliance

## Abstract

**Background:**

Long-term oxygen therapy (LTOT) extends life in patients with chronic obstructive pulmonary disease with severe hypoxaemia. Questionnaire-based studies of the effects of LTOT have not suggested uniformly positive findings. The few qualitative studies suggest that patients report benefits but also concerns about dependency on oxygen therapy. The aim of the study was to explore the views and experiences of COPD patients, their carers and the healthcare professionals who deliver these services, on the long-term use of oxygen therapy.

**Methods:**

Focus groups were conducted with 16 patients with from the LTOT service, six carers, and nine healthcare professionals (n = 31). Eleven patients with COPD, four carers and one staff manager (n = 16) participated in semi-structured interviews. Interviews and focus group were digitally recorded and field notes were taken. Data were analysed using the thematic network analysis approach.

**Results:**

Patients and carers reported the benefits of LTOT including increased social activity, perceived improvements in health status and self-management in routine daily activities. Concerns were raised regarding stigma, dependency on LTOT and deterioration in health status. Staff accounts included negative perceptions, suggesting that LTOT was often inappropriately prescribed and under-used but recommended active patient management to address this challenge.

**Conclusions:**

LTOT has some beneficial effects in improving daily activities and social interaction of patients with COPD but also some limitations. Increased support and monitoring by healthcare professionals would address some concerns, as would better information for patients, carers and the general public.

## Background

Chronic obstructive pulmonary disease (COPD) is a major cause of morbidity, mortality and healthcare utilization. It affects an estimated 210 million people worldwide and is expected to increase by more than 30% in the next ten years [1]. In 2011, the prevalence of physician diagnosed COPD in England was 1.5% and undiagnosed COPD was 2.2% of a population of 53.67 million [2], meaning that between 982,000 and 1.96 million people were living with the disease [2]. A significant proportion of COPD patients require long-term oxygen therapy (LTOT) for treatment of severe resting hypoxemia. The total cost of Long-term Oxygen Therapy (LTOT) provision in England and Wales in 2002 -2003 was over £34.8 million (US$ 52.20 million) [3].

Two landmark studies [4,5] and a Cochrane review [6] have demonstrated the benefits of LTOT on survival for COPD patients with chronic hypoxemia. Studies [7,8] that investigated the impact of LTOT on health related quality of life for patients with COPD have reported inconclusive findings. Sant’Anna and colleagues [7] found that COPD patients who were receiving LTOT had more impaired quality of life and more severe dyspnoea compared to a control group with similar characteristics. Another study [8] that investigated the relationship between male COPD patients receiving LTOT for severe hypoxemia compared with the control group in stable condition found that hypoxemic COPD patients were most likely to experience more severe dyspnoea, impaired quality of life, and elevated level of depressive symptoms compared to the control group. These patients did derive some gains, as measured by generic Health Related Quality of Life scores using the Short Form 36 item questionnaire. However, due to high prevalence of depression and excessive dyspnoea on exertion, Tsara et al., [8] suggest that COPD patients with LTOT should receive appropriate treatment for dyspnoea control and psychological support.

These studies, however, have used quantitative outcome measures such as the Medical Outcomes Study Short-Form 36-item questionnaire (SF-36). A small number of studies across a range of countries have used qualitative methods to explore the lived experience of people with COPD and their careers. Participants report physical limitations caused by their condition [9-11] causing reductions in meaningful activities [9,10,12,13] leading to reductions in social participation [9,12] and an increased feeling of dependency on their family and/or caregivers [14,15]. Depressive symptoms are also commonly reported [10,11]. This constellation of findings suggests a physical and psychological withdrawal which may be compounded by the stigmatising effect [12], self-blame [11,12] and social embarrassment [14] associated with this condition.

Specific reference to long term oxygen therapy is made in relatively few of these papers. Two studies, however, address the topic explicitly [10,15]. Whilst LTOT was viewed as beneficial, increasing dependence on it resulted in practical problems [10,15]; a particular issue for people with COPD who lived alone [15].

Three studies have reported that it was common for patients with COPD to fail to use their oxygen as prescribed by their doctors [13,16,17], whilst a further study [10] found that, whilst LTOT was deemed beneficial, increasing dependency on oxygen imposed lifestyle restrictions. Patients’ beliefs and experiences are implicated in this [18], but the area is under-researched. Listening to patients’ perspectives and tailoring services to support their expressed needs is recommended [9,14], suggesting that qualitative studies exploring patients’ views would make a valuable contribution to improve clinical practice and contribute to the current body of knowledge. Hence, this study constitutes an exploration of the views of COPD patients, their carers and the healthcare professionals who deliver these services, on the long-term use of oxygen therapy.

## Method

This study was part of a larger evaluation of services for people with COPD in one Primary Care Trust in the North of England. The data presented here represent a subsection of findings; those relating to the long term use of oxygen therapy.

### Design

An exploratory, qualitative design involving data collection using semi-structured interviews and focus groups was used to explore experiences of COPD-related health services from the perspectives of key informants.

### Participants

Participants were drawn from three groups; a client list of 461 people with a diagnosis of COPD living in a single Primary Care Trust (PCT) in the North of England; family members and other informal (i.e. unpaid) carers of the first group; and staff delivering LTOT services in the same PCT.

All service users on the client list were sent a postal questionnaire assessing levels of satisfaction with aspects of the LTOT service. A total of 189 survey questionnaires were returned, representing a response rate of 41%. Participants who responded to the survey were then invited to participate in the second (qualitative) phase of the study. We report here only the findings of the qualitative study.

Participants included those living with mild to severe forms of COPD, and varied by age and gender. All participants were currently receiving or had previously received care from the PCT’s LTOT service. The focus of the study was the various stakeholders’ experiences of using LTOT, and information was collected only by researchers from outside PCT, so individual data on COPD severity is not available. In order not to exclude potential participants with more severe COPD, however, participants were offered the option of being interviewed at home if the severity of their illness made attending a focus group too difficult. Being interviewed at home can therefore be seen as a proxy indicator of illness severity. Quotes from these participants are indicated as initials in brackets, underlined, e.g. (GR).

### Data collection

Broad topic guides were developed for the interviews and focus groups. For patients and their carers, these addressed *the impact of living with COPD*, *participants’ experiences of LTOT* and *related health service provision*. For staff, the topic guides included both the staff members’ *perceptions of service user experiences* and *their own views of the provision of the LTOT service.* Interviews were conducted by one of two research assistants skilled in qualitative interviewing (3^rd^ and 4^th^ authors). Focus groups were run by both research assistants together.

Four service user focus groups were conducted comprising a total number of 22 participants; 16 people with COPD and six informal carers. The focus groups took place at three community locations to ensure convenience and optimum involvement.

Eleven in-depth, semi-structured interviews were conducted with service users in their homes where possible or over the telephone when more convenient. Four semi-structured interviews completed with informal carers of people with COPD.

Two focus groups were conducted with staff employed as part of the COPD LTOT team. A total number of nine staff attended the two focus groups. Participants had varying levels of expertise of working in the LTOT service, representing a broad cross section of staff including respiratory nurse specialists (n = 4), a respiratory support nurse, a senior physiotherapy respiratory specialist, a physiotherapy assistant, a clinical support worker, and a support secretary. One additional interview was conducted with the manager of the COPD LTOT service.

### Data analysis

The interview and focus group data were transcribed verbatim. All references relating to long term oxygen therapy were entered into NVIVO qualitative data analysis software and analysed following the thematic network analysis approach (TNA) [19]. TNA is increasingly being used for analysis of interview and focus group data in health and social care settings [20-24] and is recognised as a robust form of thematic analysis [25], particularly useful in identifying and portraying hierarchies of themes.

Data analysis followed the prescribed procedure [19], p.391. The first stage involved open coding using NVIVO. Coding was inductive, and represented recurrent and salient issues within the text. The codes were applied to meaningful segments of the textual data.

Stage two involved re-reading of text segments within each code, identifying the underlying pattern and hence, abstracting “Basic Themes.” The third step involved bringing together the Basic Themes and grouping them into Organizing Themes (OTs) which summarized the main assumptions of a group of BTs and are thus more abstract. OTs also reflects distinct main assumptions of the super-ordinate Global Themes, which represent the major points in the focus groups and interviews. This network can be used to discuss the research questions.

### Quality issues

In fields where qualitative research is relatively new, there is an onus on researchers to be explicit about the processes they have undertaken to ensure the rigour and robustness of their findings. In this study two recognised approaches were used; peer review of coding and peer debriefing [26]. Peer review of coding took the form of comparing the themes presented in this paper, which was conducted by the first author, with coding of data relating to LTOT in a report on the larger study conducted by the third author. Peer debriefing involved detailed discussion of the process and findings between the first author and the second author, an authority on COPD, to ensure that there was a clear audit trail from the transcripts, through the basic themes and organizing themes to the global themes.

### Ethical issues

The study was subjected to ethical scrutiny at the first author’s university and given ethical approval by the relevant Faculty Academic Ethics Committee. The proposed study was discussed with the Trust R&D department who approved it as “service evaluation”.

## Results

Long term oxygen therapy was discussed in five (of 11) individual interviews with service users and all four focus groups of services users and informal carers. It was also raised in (the) one staff interview and one (of two) staff focus groups. As the focus groups with service users and family carers were conducted jointly, with both sets of participants contributing to and discussing ideas, their responses are analysed together.

**Figure 1:**
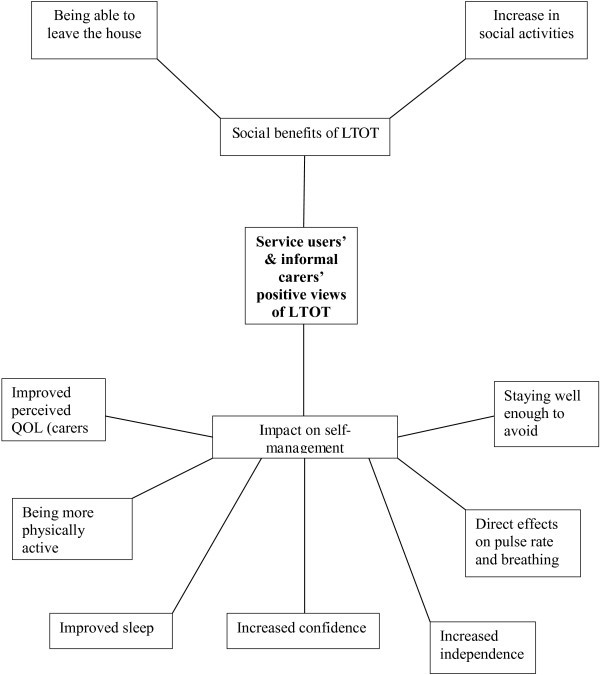
Global theme: service users’ and informal carers’ positive views of LTOT.

Two global themes emerged for service users and informal carers: *positive views of long term oxygen therapy* and *negative views of long term oxygen therapy.* In contrast, only one global theme emerged from the staff: *professional roles and LTOT.* The resulting networks of Global Themes, their constituent Organizing Themes and contributing Basic Themes is presented in Table 1 and the network for the Global Theme *positive views of long term oxygen therapy* is portrayed in Figure 1.

**Table 1:** Hierarchy of emergent themes

**Basic themes**	**Organising themes**	**Global themes**
Being able to leave the house	*Social benefits of LTOT*	**GT1: Service users’ &informal carers’ positive views of LTOT**
Increase in social activities		
Improved perceived QOL (mentioned by carers only)	*Impact on self-management*	
Staying well enough to avoid hospital		
Being more physically active		
Direct effects on pulse rate and breathing		
Improved sleep		
Increased confidence		
Increased Independence		
Own views of the equipment	*Negative views concerning the LTOT equipment*	**GT2: Service users’ &informal carers’ negative views of LTOT**
Others’ views of the equipment		
Progression of the disease despite the benefits of LTOT	*Concerns regarding deterioration in health status and dependence on LTOT*	
Increasing dependence on LTOT		
LTOT as an unused resource	*Staff negative views of other professionals re LTOT*	**GT3: Professional roles and LTOT**
Inappropriate prescription of LTOT		
Intervention to enhance the use of LTOT	*Active patient management*	
Multidisciplinary reviews		
Other rehabilitation strategies		

Each global theme will now be presented; the constituent organizing themes and contributing basic themes will be described and illustrated by quotes. The source of the quotes is given in brackets, with interview respondents identified by anonymised initials for the carers and anonymised initials underlined for the interviewees with COPD.

### Global theme 1: positive views of LTOT: service users and informal carers

Positive comments were made in all four Focus Groups and in four of the five individual interviews. Two organizing themes contributed to the above global theme; social benefits of LTOT and impact on self management.

### Social benefits of LTOT

Service users and their carers identified benefits to their social lives as a significant benefit of LTOT. Two Basic Themes were specified; being able to leave the house and a related increase in social activities.

In contrast with their previous experience of feeling trapped at home, participants valued the flexibility offered by portable oxygen therapy systems that enabled them to leave the house.

*I can get out during the day and at night. It has made all the difference.* (Focus Group 3)

*I can go on days out and things like that which I couldn’t do before.* (Focus Group 4)

*You can get out and breathe outside so you don’t have to stay in.* (Focus Group 3)

As a consequence of being able to leave the house, participants and their carers were also experiencing and improvement in their social lives.

*I can go on days out and things like that which I couldn’t do before because if we did it would only be from A to B and not do anything, I would sit in the car whilst they were doing things ….. whereas now it means that I can go on a day out and go with them where they’re going so that has made a big difference.* (Focus Group 4)

*I take an elderly auntie, she’s 80 and I can take her out maybe once a week to a garden centre or something, for lunch, and I go to lunch with one of my friends.* (PD)

### Impact on self management

Service users and their carers identified a range of ways in which they felt better able to manage their daily lives in the context of the physical and psychological implications of COPD. Seven basic themes contributed to this organizing theme. Of these, one; improved perceived quality of life (QOL) was mentioned by carers only. The remaining basic themes, which may reflect contributing aspects of QOL, staying well enough to avoid hospital, being more physically active, direct effects on pulse rate and breathing, improved sleep, confidence and independence, emerge from the responses of both groups.

In response to specific questions, participants reported a reduction in hospital admission since starting LTOT.

I: Have you been into hospital at all since you’ve been on the oxygen, been involved with the service?

C: Oh yeah, yeah. Yeah, but I haven’t … three times I’ve been in … but this is the longest time … you know.

I: The longest that you’ve not been in?

*C: Yeah … touch wood!* (CJ).

Service users and their carers reported a positive effect on physical activity levels.

*Walking from here to do the door without the oxygen I’d be on my knees but with the oxygen I can walk that little bit further.* (Focus Group 2)

*I can walk a longer distance.* (Focus Group 2)

*But he is standing up straight now and he is just generally more alert.* (JV)

Specific impact on pulse rate and breathing was also identified.

*It is so easy to take your own pulse and it is 120 sometimes but after the oxygen for half an hour it starts climbing down again and that is the major thing.* (JV)

Service users and their carers reported improved sleeping which was beneficial for both parties.

*Prior to that I would about 1 or 2 hours and now I sleep all night so that’s made a big difference.* (Focus Group 4)

*When she goes to bed she hasn’t got that fear. She was waking. Say 8 months ago I was doing a 23 hour day. She was waking up screaming that she was dying because she couldn’t breathe but since she has been on the oxygen thing which I try to keep as minimum as possible. I keep it down as much as I can, she goes to bed and I give her tablets and luckily now I am getting through to 6 in the morning.* (Focus Group 3)

More diffuse benefits in the form of increased confidence and independence were mentioned by users of LTOT and their careers. These were seen as distinct from physical benefits, but just as welcome.

*It does give you confidence, regardless of what it is physically doing for you.* (Focus Group 3)

*I feel as though I’ve got some independence back whereas before I was 100% reliant on other people doing things.* (Focus Group 4)

*He will make a cup of tea now and that is a big step for us. I know it doesn’t seem very much but it is a big step.* (JV)

Increased quality of life could be seen as a general category encompassing many of the other perceived benefits to participants’ self-management of their condition consequent on LTOT. The specific term however, was mentioned only by carers.

*It is not going to change his life but it has picked him up. It has given him a better quality of life.* (JV)

### Global theme 2: negative views of service users and informal carers

Not all aspects of being on long term oxygen therapy were perceived as benefits by service users and their informal carers. Two organizing themes emerged; negative views concerning the LTOT equipment and concerns regarding deterioration in health status and dependence on LTOT.

### Negative views concerning the LTOT equipment

These views were expressed in relation to the equipment *and* in relation to others’ views of the equipment.

*Me social life’s just gone. I can’t go out or anything you know, because I’ve gotta drag this with me.* (GV)

*Before we knew it this tank was there, I mean I got a shock when I walked in so it was a shock for me so I can’t imagine how poor mum felt.* (Focus Group 2)

Other people’s judgements seemed to be operating against the positive benefit of increased social activity.

*I never wore it to go out because I was too embarrassed.* (Focus Group 1)

*You get people looking at you and saying oh aren’t you brave coming out.* (Focus Group 4)

*The secretary of the ….. Club doesn’t like me erm, he doesn’t like me wearing it*. (GV)

The progressive nature of the disease and dependency, and the fear of these, could be seen as inevitable consequences of COPD. A sense of the progression of the disease despite the benefits of LTOT was reported by service users and carers.

*I have oxygen at home but it is difficult, well I am not getting out at all now.* (Focus Group 3)

*Now he is just not fit enough to do anything.* (SM)

Concerns regarding deterioration and dependence on. LTOT

Although the advantages of LTOT were well recognized, there was a perception that its use implied dependence and that this was a negative step.

*I have kept not wanting it really because it takes away your independence.* (Focus Group 3)

### Views of staff: global theme 3: professional roles and LTOT

Interview and focus group data from participating members of staff tended to address practical aspects relating to their own and other professionals’ roles in the management of patients with COPD. Two organizing themes contributed to this; staff negative views of other professionals re LTOT and active patient management.

### Staff negative views of other professionals regarding LTOT

Staff members tended to be critical of the ways in which LTOT was being used. Two basic themes emerged from the data; LTOT as an unused resource and inappropriate prescription of LTOT.

Staff expressed concern at the perceived wastage of oxygen therapy equipment prescribed to service users which was not used. Although specific numbers were not cited, staff seemed to feel that this was a frequent occurrence.

*Most people who get portable oxygen, they just sit and look at it. It is never used and I think that is probably one of the most wasted resources really.* (interview)

*In some cases, oxygen equipment had just been lying in homes without being used.* (Focus Group – Staff)

A related issue was inappropriate prescription of LTOT. This included staff members’ concerns regarding over-use and view that service users lacked information which would support appropriate use.

*There are a lot more who could stop it but they are frightened to let it go and we are not here for that reason. Some people didn’t know how to use it really, it had been tagged on to the end of a prescription and they didn’t really know what it was really for or it was too heavy for them to use but nobody knew.* (interview)

*Some people had been overusing oxygen. Others were really struggling with oxygen, but not using the oxygen because they did not know what sort of relief was going to be provided.* (Focus Group – Staff)

### Active patient management

Rather than accept this situation, staff members saw their role as active patient management, taking responsibility for improving the way LTOT was being used by patients, but also influencing the service provided by professional colleagues. This could include interventions to enhance the use of LTOT as well as multidisciplinary reviews and other rehabilitation strategies.

*Also if we pick up patients who are using their oxygen because they are perhaps under-medicated and they need a review, they need to be supported. That is how we are picking up patients who are using oxygen because there is a gap in their management somewhere so we are passing patients across for COPD review or for pulmonary rehabilitation.* (interview)

*So it is about going in there, educating them, putting things in place to better support them. The service is about the long-term.* (Focus Group – Staff)

## Discussion

The main findings the study include: 1) Patients and carers have reported the benefits of LTOT in increased social interaction, perceived health status and self-management in routine daily activities; 2) COPD patients and carers have raised concerns regarding dependency on LTOT and deterioration in their health status; and 3) The staff reported concerns regarding the inappropriate prescription of LTOT and their perception that LTOT was not being used as prescribed. They were, however, pursuing active strategies to ameliorate this situation.

### The views of people with COPD and their carers

The balance of evaluative comments from service users and informal carers comes down very much in favour of long-term oxygen therapy. In particular, by enabling people to leave their homes, portable systems are seen as facilitating the resumption of social activities. Whilst this could be seen as participants giving socially desirable responses, the content of the quotes supports the interpretation that most patients with COPD and their carers derive benefit from LTOT. This contrasts with the experiences of Godoy et al (2012)’s participants [13], the majority of whom, despite using LTOT, were unable to participate in leisure activities. Measurable indicators of improved physical health status, such as reduced hospital admissions, reduced pulse rate, increased sleep duration, were given by many participants in the current study, allowing participants to feel they were managing their condition better, further supporting the perceived benefits of LTOT.

The experiences of some participants, however, echoed the negative spiral of physical limitations causing reduced activities and participation, and consequent implications for mental health reported in a number of studies [9-12,14,15]. These problems seem to be exacerbated by negative feelings about the equipment required to deliver LTOT, and stigma and social embarrassment [12,14]. There are implications here regarding the guidance required by patients and carers, but also a suggestion that public awareness campaigns regarding the use and value of portable oxygen equipment could be beneficial.

### The views of staff

The lack of explicit positive evaluations by staff members is interesting, but it may be that they take the advantages of LTOT as given, or they would not be party to its continued prescription. It is clear, however, that healthcare professionals involved in this study consider LTOT, at times, to be inappropriately prescribed and, even where appropriate, frequently under-used. As Godoy et al (2012) suggest [13], there is a need for healthcare staff to undertake regular domiciliary visits to patients prescribed LTOT in order to ensure that it is being used, and used correctly. This might alleviate some of the concerns expressed by participants with COPD regarding deterioration of their condition and dependence on LTOT.

### Limitations of the study

First, participants were self-selected, meaning that those with stronger views might have been more likely to volunteer. Inclusion of interviews at home facilitated the inclusion of people with severe COPD. It should not be forgotten, however, that patients with COPD can be very unwell, and the views of those with very severe illness may have been under-represented. Second, the participation of both patients and carers in the same focus groups meant that it was not always possible to disaggregate the views of the two groups. This requires further investigation.

Third, whilst the views of patients interviewed at home (those with more severe COPD) do not seem to differ from those of the focus group participants, a formal analysis of responses by disease severity was not possible, because lung function and other measures of severity were not measured.

Finally, whilst this study can be regarded as a useful and innovative approach to capturing the complementary perspectives on LTOT of patients and carers, and healthcare professionals in one location, it needs to be repeated across different geographical locations with differing service delivery models.

### Implications of the study

This study has identified a number of service implications to improve clinical practice. The value placed by patients with COPD and their carers on the additional independence and access to a social life that LTOT offers is clear. This is counterbalanced, however, by their concerns about dependence on LTOT and those of the healthcare professionals that LTOT is often not prescribed or used appropriately. To maximise the benefit gained by patients, there is a need for regular monitoring and support from health care professionals. It is important that patients and carers receive appropriate education in the use of LTOT to optimise usage and reduce fears and barriers to oxygen treatment. Healthcare professionals and LTOT providers should be vigilant in removing equipment if no longer required due to patients’ improved health status. In addition, there is a need raise public awareness of the safety and value of portable oxygen equipment and the benefits it offers.

## Conclusion

LTOT is perceived by service users as offering some beneficial effects in improving self-management and social activities for patients with COPD. Further research with service users, carers and healthcare professionals is needed to maximise the appropriate use of LTOT services.

## Competing interests

The authors declare that they have no competing interests.

## Authors’ contributions

JG designed data collection and analysis approaches for the qualitative study, analysed the LTOT data, participated in the peer review of coding against full data set and engaged in a peer debriefing exercise with AMY. AMY designed the over-arching study, provided theoretical and research background on COPD and conducted the peer debriefing with JG. RW conducted interviews and focus groups, and conducted independent analysis of full data set enabling peer review of coding. SC conducted interviews and focus groups, and contributed to early stages of analysis. All authors read and approved the final manuscript.
